# Assessment of college students’ awareness and knowledge about conditions relevant to metabolic syndrome

**DOI:** 10.1186/1758-5996-6-111

**Published:** 2014-10-15

**Authors:** Najat Yahia, Carrie Brown, Melyssa Rapley, Mei Chung

**Affiliations:** Department of Human Environmental Studies, Central Michigan University, Wightman 108, Mt. Pleasant, MI 48859 USA; Department of Public Health and Family Medicine, Tufts University School of Medicine, 136 Harrison Avenue, Jaharis 264, Boston, Massachusetts 02111 USA

**Keywords:** Metabolic syndrome, Cardiovascular risk factors, Adiposity, College students, Lay knowledge

## Abstract

**Background:**

Despite the increasing prevalence of metabolic syndrome among young adults, little is known about the awareness level of college students about this condition. The purpose of this study was to assess students’ level of awareness and knowledge about conditions relevant to metabolic syndrome (MetS).

**Methods:**

A self-reported online questionnaire was administered to 243 students attending Central Michigan University. Questions were divided into seven conditions: diabetes, adiposity, hypertension, high serum cholesterol, arteriosclerosis, stroke, and myocardial infarction. Students’ responses were scored and interpreted as follows: *poor knowledge* if ≤50% of students answered the question correctly; *fair knowledge* if between 51-80% of students answered the question correctly; and *good knowledge* if between 81-100% of students answered the question correctly. Anthropometric measurements including height, weight, waist circumference, percentage body fat, and visceral fat score were measured. Fisher’s exact test was used to test the differences in students’ responses. A *p* value <0.05 was considered a statistically significant difference.

**Results:**

More than 80% of students correctly identified symptoms and complications of diabetes, hypertension, arteriosclerosis, myocardial infarction and stroke, and 92% identified adiposity as a risk factor for heart disease. There were few false beliefs held by students on questionnaire items. For example, 58% of male students falsely believed that individuals with diabetes may only eat special kinds of sweets compared to 39% of females (*p* < 0.01) and more than half of the students falsely identified liposuction as the best possible treatment in adiposity therapy. Gender, Health Science major, and year in school were found to be positively associated with more knowledge.

**Conclusion:**

The findings in this study suggest that students’ knowledge about conditions relevant to metabolic syndrome can be improved. In this essence, raising awareness about MetS based on students’ pre-existing knowledge is essential to enhance students’ wellness.

## Background

Metabolic syndrome (MetS) is a constellation of interrelated cardio-metabolic risk factors that include central obesity, hyperglycemia, hypertension and dyslipidemia [1–4]. Individuals with MetS are at increased risk for type 2 diabetes [5], cardiovascular disease (CVD) [6, 7], and all-cause mortality [8]. According to the National Cholesterol Education Program’s Adult Treatment Panel III (NCEP ATP III) report, MetS is identified in individuals when at least three of the following five risk factors are present simultaneously: increased waist circumference, elevated blood pressure, elevated serum triglyceride, reduced HDL cholesterol (HDL-C), and elevated fasting plasma glucose [9]. The underlying cause of this syndrome is not yet known, but its manifestation has been linked to obesity (especially abdominal obesity) and insulin resistance [10].

Current literature indicates that MetS is prevalent [11–14] and it is increasing over time [15]. Estimates from the 2003–2006 National Health and Nutrition Examination Survey (NHANES) suggest that 34% of U.S. adults aged 20 years and over have MetS [13]. This represents a 6% increase in the prevalence of MetS from the 1999–2000 NHANES data [15]. Alarmingly, the prevalence of MetS is common not only among U.S. adults but also among younger persons, and is increasing in parallel with a rise in central obesity [15–17]. According to NHANES, 20.3% of males and 15.5% of females, aged 20–39 years, have MetS [13]. Likewise, MetS prevalence was also documented among college students, in the range of 0.6% to 13% [18–21]. Results from the Young Adult Health Risk Screening Initiative study, conducted among 2722 college students, aged 18–24 years, indicated that 10% of males and 3% of the female participants have MetS [22]. Further, the authors reported that 77% of the males and 54% of the female participants displayed at least one risk factor for MetS (elevated blood pressure in men and low concentrations of HDL-C in women) [22]. Similarly, a study conducted among 300 students at the University of Kansas found that 26% to 40% of the students had at least one metabolic risk factor (low concentrations of HDL-C) [19].This is critical as risk factors for MetS are known to increase the risk of CVD and diabetes later in life. In fact, individuals with MetS appear to have a two-fold increase in risk for CVD and a four-fold increase in risk for type 2 diabetes [22]. Thus, early risk factor detection and intervention is essential to prevent or reduce the development of MetS and, eventually, cardiovascular health problems later in life [23–26].

Universities are unique settings for early risk factor detection and intervention so appropriate lifestyle changes can be implemented at an early age. However, prior to developing any prevention or intervention strategy, assessing the level of awareness and knowledge of conditions relevant to MetS among students is essential. According to the Health Belief Model, risk perception is a primary motive to change a behavior, and the greater the perceived threat, the more likely an individual will change his/her behavior [27, 28]. In this essence, college students should first be aware of MetS and then must perceive themselves at risk so appropriate lifestyle choices can be made. However, a lack of awareness/knowledge might hinder such a change. As such, students who are unaware of MetS or lack knowledge about MetS may not perceive themselves at risk for this condition and, consequently, may go undiagnosed until cardiovascular and diabetes complications occur. Thus, assessing students’ level of awareness and knowledge about conditions relevant to MetS is vital to developing any prevention strategy to reduce MetS.

Currently, there is very little information in the literature about studies examining students’ knowledge of conditions relevant to MetS. Current literature indicates that most college students are unaware of MetS or CVD risk factors and that some students hold false beliefs about CVD complications [29, 30]. Previous reports have indicated that college students do not accurately perceive their own risk factors and rate their own risks lower than their peers’ risk [31]. Thus, the main objective of this study was to assess awareness and knowledge of conditions relevant to MetS among a sample of students from Central Michigan University (CMU). The outcomes of this study may have implications for the design of tailored health promotion programs based on students’ pre-existing knowledge to improve students’ overall health.

## Methods

### Design and sample

This study was a cross-sectional survey. Out of 270 students recruited, 243 students (174 females and 69 males) completed all the required study assessments, yielding a response rate of 90%. Students were recruited randomly, both in Foods and Nutrition classes and via Blackboard announcements, by a CMU Nutrition and Dietetics professor during fall 2011 and spring 2012 semesters. Students voluntarily entered the study and were provided adequate information about the study protocol. Students agreeing to participate were asked to sign a consent form, in harmony with the Helsinki declaration, and then to come to a laboratory for anthropometric measurements and to receive a numerical code for completing a self-administered online questionnaire. Students were not offered any incentives for their participation. The study protocol was approved by the CMU Institutional Review Board (IRB).

### Data collection

Data collection took place in two steps. First, students’ anthropometric measurements including height, weight, waist circumference, percentage body fat, and visceral fat score were measured. Weight, percentage body fat, visceral fat score, and body mass index were determined by *Tanita* scale body fat analyzer 300A. As fluctuations in body hydration status may affect body composition results, students were instructed to fast and to refrain from any heavy physical activity before *Tanita* scale measurements were taken in the morning (within 3 hours after waking up). In addition, students were asked to wipe off the bottom of their feet before stepping onto the measuring platform, since unclean foot pads may interfere with conductivity. Height measurements were taken with a stadiometer. Students were asked to take off their shoes for height measurements. Body mass index (BMI) was used to assess students’ weight status [32]. Normal ranges for percentage body fat were considered as follows: 10-20% for males and 20-30% for females based on *Tanita* score values. In the second step, students were asked to complete a self-administered online questionnaire consisting of questions related to students’ demographics and characteristics, and 90 questions about students’ knowledge about conditions relevant to MetS. The demographic and student characteristics questions were adapted from Yahia et al. [33]. The knowledge questions were adopted from a previous study by Becker et al. [29]. These questions were tested, standardized and validated to be used among university students in a previous study [29]. In addition, the selected adapted questions were administered to a pilot group of 20 students to ensure adequate understanding of the items.

The 90 questions about students’ knowledge were about the conditions often leading to MetS, the outcome and treatment of these conditions, and a description of physical changes relevant to MetS. The questions were divided into seven categories: diabetes (16 questions), adiposity (9 questions), hypertension (12 questions), high serum cholesterol (6 questions), arteriosclerosis (17 questions), stroke (12 questions), and myocardial infarction (18 questions). The response options to the questions were “true”, “false”, or “do not know”. Students’ responses were scored. The “correct” response was awarded one point and the “incorrect” and “do not know” responses were awarded zero points. The maximum possible total score for the MetS questions was 90. Prior to taking the questionnaire, each student was given a numerical code to use. The purpose of coding was to maintain anonymity of the students and to allow them to answer the questions honestly without being identified. The questionnaire was a web-based survey administered via *Survey Monkey* and was available online for about 10 weeks to accommodate students’ response times.

The definitions for the levels of students’ knowledge were as follows: *poor knowledge* if ≤50% of students answered the question correctly; *fair knowledge* if between 51-80% of students answered the question correctly; and *good knowledge* if between 81-100% of students answered the question correctly [34]. In this study, the questionnaire was pilot-tested on a randomly selected group of 20 students prior to its administration. Students were given instructions on how to fill out the questionnaire completely and truthfully and to skip a question if unsure of the answer.

### Data analysis

All analyses were performed using the SAS® 9.3 (U.S.A.) software. Descriptive analyses were performed for all study participants. Student’s t-test was used to examine differences in the continuous variables (age, weight, height BMI,% body fat, visceral fat, and waist circumference) between male and female students. Fisher’s exact test or chi-squared was used for the categorical variables (Health-Science vs non-Health Science majors of study, year in school, and presence of family history of various diseases). Fisher’s exact test was also used to examine differences in students’ responses to the questionnaire.

To examine the associations between several variables (such as gender, major of study, year in school, family history, weight status, and ethnicity) and total score for the MetS questions, a Poisson regression model was used. In this model, total score was used as the *dependent variable* and gender, major of study, family history (any), overweight/obesity, ethnicity, and year in school, were used as *independent variables*. To assess how these variables were associated with scores on the seven different sections of the questionnaire, seven t-tests were performed to test the differences in the percentage of correct answers for each section (to standardize the unit across the seven sections because the maximum score across the seven sections varied) by the following categorical variables: gender, major, any family history, overweight/obese, ethnicity, and year in school. For all tests, *p* < 0.05 was used to determine significance; however, since there are multiple tests, this analysis should be considered exploratory. Results were expressed as means ± SD (standard deviation). All reported *p* values were 2-sided and a *p* value less than .05 was considered statistically significant.

## Results

### Participants’ characteristics

A sample of 243 students (72% females and 28% males), with a mean age of 20.6 years, participated in this study. The average weight and height of the participating students were 69.8 ± 16.7 kg and 166.8 ± 9.1 cm, respectively. The mean BMI and percentage body fat for males were 26.1 ± 4.9 kg/m^2^ and 16.6 ± 8.1, respectively, whereas for females values were 23.4 ± 3.9 kg/m^2^ and 26.8 ± 6.9, respectively. The mean values of visceral fat scores for males and females were 1.9 ± 1.8 and 3.7 ± 3.4, respectively (Table 1). Of the participating students, 89% were Caucasian, 10% African American, and 1% other ethnicity, reflecting the composition of the ethnic groups at CMU. More than half of the students were Health-Science major (51%) and most of them were in their second and third year of academic study. More women reported a science major (58%) than men (36%). Smoking was not common among students, as 89% of students reported being non-smokers, 6% were current smokers, and 5% were former smokers. Family history of chronic diseases among students was distributed as follows: 13% heart disease, 23% diabetes, 41% hypertension, and 26% cancer. This demographic profile is consistent with national averages in regard to students’ age, gender, and year in school [35], but different in regard to less ethnic diversity, lower percentage of smokers [35], and less obesity [24].

**Table 1:** **Demographics and student characteristics (Means ± SD)**

Variables	Females	Males	Total
**Number of students**	N =174	N =69	N =243
**Age (Years)**	20.4 ± 1.7*	21.3 ± 2.4*	20.6 ± 2.0
**Weight (Kg)**	64.1 ± 12.7*	84.2 ± 17.0*	69.8 ± 16.7
**Height (cm)**	162.9 ± 6.6*	176.0 ± 6.7*	166.8 ± 9.1
**BMI (kg/m** ^**2**^ **)**	23.4 ± 3.9*	26.1 ± 4.9*	24.2 ± 4.4
**Body fat (%)**	26.8 ± 6.9*	16.6 ± 8.1*	23.9 ± 8.6
**Visceral fat score**	1.9 ± 1.8*	3.7 ± 4.2*	2.4 ± 2.8
**Waist circumference (cm)**	81.0 ± 9.7*	91.2 ± 11.0*	83.6 ± 11.0
**Major of study (%)**			
Health science**	58%*	36%*	51%
Non-health science	42%*	64%*	49%
**Year in school** (%)			
1^st^-year undergraduate	14%	15%	15%
2^nd^-year undergraduate	27%	21%	25%
3^rd^-year undergraduate	27%	25%	27%
4^th^-year undergraduate	19%	19%	19%
5^th^-year undergraduate	13%	19%	15%
**Ethnicity (%)**			
White (Hispanic & Non-hispanic)	91%	86%	89%
Black (Hispanic & Non-hispanic)	9%	10%	10%
Other	0%	4%	1%
Family history of chronic disease (%)***			
Heart Disease	10%*	20%*	13%
Diabetes	23%	25%	23%
Hypertension	39%	41%	41%
Cancer	12%	61%	26%
Others****	44%*	21%*	38%
**Smoking habit (%)**			
Non- smoker	93%*	80%*	89%
Current smoker	3.5%	12%	6%
Former smoker	3.5%	9%	5%

*p < 0.05 (between male and female students).

**Health Science majors include: Exercise Science, Health Administration; Health Fitness in Preventive and Rehabilitative Programs, Public Health Education and Health Promotion, School Health Education, Exercise Science, Personal and Community Health, Substance Abuse Education, Prevention, Intervention and Treatment.

***Total% exceeds 100% due to students with a family history of more than one condition.

****Other - includes the “other” *family history questions* in the data such as family history of overweight, obesity, and high cholesterol.

### Metabolic syndrome questionnaire

Overall, results show that students were most knowledgeable about the two conditions: arteriosclerosis and stroke conditions, and least knowledgeable about adiposity and cholesterol. Table 2 shows items where more than 80% of the students answered the questions correctly- indicating *good knowledge* on questionnaire items; Table 3 shows items where 51-80% of the students answered the questions correctly - indicating *fair knowledge*; and Table 4 shows items where ≤50% of the students answered the questions correctly- thus indicating *poor knowledge* or areas for improvement.

**Table 2:** **Students’ response to the MetS questionnaire by gender -** ***good*** **level of knowledge - (percentage of students answered each question correctly between 81-100%)**

Conditions	Question	Correct answer	***Males (N = 69)***		***Females (N = 171)***		***Total (N = 240)***		***P value***
			***n***	***%***	***n***	***%***	***n***	***%***	
***Diabetes***	There are several different types of diabetes.	**True**	62	90.0	144	84.2	206	85.8	0.310
	Pregnant women have a reduced risk of acquiring diabetes.	**False**	56	81.2	148	86.6	204	85.0	0.320
	Eye disorders can be consequences of diabetes.	**True**	60	87.0	134	78.8	194	81.2	0.201
***Adiposity***	Adipose individuals have an elevated risk of suffering myocardial infarction.	**True**	63	91.3	15	92.9	219	92.4	0.788
	Cessation of breathing while sleeping is a possible consequence of adiposity.	**True**	52	75.4*	147	87.5*	199	84.0	0.031
	Adipose individuals are more likely to suffer from arteriosclerosis.	**True**	53	76.8	146	86.9	199	84.0	0.078
***Hypertension***	Hypertension can cause dizziness.	**True**	60	88.2	144	85.2	214	86.1	0.679
***High serum cholesterol***	A low cholesterol diet can supplement therapy for high serum cholesterol.	**True**	62	89.9	148	89.2	210	89.4	1.000
	High serum cholesterol can be treated with medication.	**True**	58	84.1	142	85.5	200	85.1	0.841
	High serum cholesterol promotes arteriosclerosis.	**True**	53	76.8*	145	87.9*	198	84.3	0.046
***Arteriosclerosis***	Arteriosclerosis increases the risk of suffering a stroke.	**True**	59	85.1*	162	96.4*	221	93.3	0.007
	Leg pains are a symptom of arteriosclerosis.	**True**	56	81.2	148	88.1	204	86.1	0.214
	In arteriosclerosis, a sustainer can be inserted into the artery in order to stabilize it.	**True**	56	81.2*	151	91.0*	207	88.1	0.041
	Individuals with high blood pressure are more likely to suffer from arteriosclerosis.	**True**	50	72.5*	148	89.2*	198	83.9	0.003
***Stroke***	A stroke affects the brain.	**True**	61	88.4*	162	97.0*	223	94.5	0.023
	If a patient survives a stroke, there are usually no permanent consequences.	**False**	52	75.4*	146	88.0*	198	84.3	0.011
	Permanent speech defects are possible consequences of a stroke.	**True**	59	85.5*	159	95.1*	218	92.4	0.015
	A stroke is often followed by memory dysfunction.	**True**	52	75.4*	150	89.8*	202	85.6	0.007
	There are different types of strokes.	**True**	54	78.3*	151	90.4*	205	86.9	0.018
	A stroke is preceded frequently by speech problems.	**True**	58	84.1	146	88.0	204	86.4	0.407
***Myocardial infarction***	When suffering a myocardial infarction, pain may radiate into the arms.	**True**	58	85.3	155	93.4	213	91.0	0.075
	Hereditary factors play a role in the risk of suffering a myocardial infarction.	**True**	51	75.0	138	83.6	189	81.1	0.142
	After a myocardial infarction, anticoagulants are administered.	**True**	60	88.2	148	89.2	208	88.9	0.822
	A myocardial infarction is often preceded by shortness of breath.	**True**	54	79.4*	153	92.2*	207	88.5	0.012
	A myocardial infarction is caused by arterial obstruction.	**True**	53	77.9	146	88.0	199	85.0	0.068
	After a myocardial infarction has occurred, parts of the cardiac muscle tissue can die.	**True**	64	92.6	158	95.8	222	94.9	0.344
	With a myocardial infarction, cardiac muscle tissue dies.	**True**	51	75.0	141	84.9	192	82.1	0.091

*p < 0.05.

**Table 3:** **Students’ response to the MetS questionnaire by gender –** ***fair*** **level of knowledge – (percentage of students answered correctly between 51-81%)**

Conditions	Question	Correct answer	***Males (N = 69)***		***Females (N = 171)***		***Total (N = 240)***		***P Value***
			***n***	***%***	***n***	***%***	***n***	***%***	
**Diabetes**	Hereditary factors play a major role in the development of diabetes.	**True**	54	78.3	138	80.7	192	80.0	0.722
	An increased alertness is a frequent symptom of diabetes.	**False**	47	68.2	141	82.5	188	78.3	0.310
	Hereditary factors play only a minor role in the development of diabetes.	**False**	43	62.3	112	65.5	155	64.6	0.657
	For some individuals with diabetes it is not advisable to take insulin.	**True**	57	82.6	119	69.6	176	73.3	0.052
	Individuals with diabetes may only eat *special* kinds of sweets for diabetes.	**False**	29	42.0*	104	60.8*	133	55.4	0.010
	With diabetes, sugar cannot enter the cells sufficiently.	**True**	47	69.1	103	60.6	150	63.0	0.237
	Poor appetite is a frequent symptom of diabetes.	**False**	33	47.4	93	54.4	126	52.5	0.393
	With diabetes, too much sugar enters the cells.	**False**	45	65.2	89	52.1	134	55.8	0.084
	Pregnant women have an increased risk of acquiring diabetes.	**True**	47	69.1	134	78.4	181	75.7	0.136
	Frequent urination is a classic symptom of diabetes.	**True**	55	79.7	121	71.2	176	73.6	0.198
	Arteriosclerosis is one of the sequalae of diabetes.	**True**	44	63.8	109	64.1	153	64.0	1.000
***Adiposity***	An excessively fatty, high-caloric is the only factor that determines adiposity.	**False**	43	62.3	105	62.5	148	62.5	1.000
	Adipose individuals have the same risk than non-adipose individuals of suffering a stroke.	**False**	45	65.2	120	71.4	165	69.2	0.355
	Adiposity can be treated surgically.	**True**	45	65.2	128	76.2	173	73.0	0.107
***Hypertension***	Hypertension is associated with heredity.	**True**	54	79.4	138	80.7	192	80.3	0.858
	For the most part, a concrete single reason of why a patient suffers from hypertension can be determined.	**False**	33	48.5	103	60.2	136	56.9	0.067
	Pregnant women are less likely to suffer from hypertension.	**False**	48	70.6*	143	83.6*	191	79.9	0.031
	After medication has lowered hypertension, the medication can usually be discontinued.	**False**	44	64.7	115	67.7	159	66.8	0.761
	Individuals with hypertension are less likely to suffer from arteriosclerosis.	**False**	44	64.7*	134	78.8*	178	74.8	0.031
	Hypertension can be caused by disorders of the thyroid gland.	**True**	54	79.4	133	78.4	187	78.6	1.000
	Hypertension can cause renal damage.	**True**	53	77.9	134	78.8	187	78.6	0.863
	Hypertension can lead to eye disorders.	**True**	49	72.1	119	70.0	168	70.6	0.875
	Hypertension can be caused by cerebral tumors.	**True**	43	63.4	111	65.3	154	64.7	0.766
***High serum cholesterol***	High serum cholesterol is not associated with hereditary factors.	**False**	46	66.7*	133	80.6*	179	76.2	0.028
***Arteriosclerosis***	With arteriosclerosis, arteries become softer.	**False**	42	60.8	109	64.9	151	63.7	0.556
	Arteriosclerosis can be cured completely.	**False**	48	69.6	135	80.4	183	77.2	0.088
	With arteriosclerosis, arteries become less elastic.	**True**	45	65.2*	141	83.9*	186	78.5	0.003
	As a result of arteriosclerosis, blood pressure is likely to decline.	**False**	42	60.9*	129	76.8*	171	72.2	0.017
	As a result of arteriosclerosis, blood pressure is likely to increase.	**True**	53	76.8	136	81.0	189	79.8	0.480
	High blood pressure and arteriosclerosis are not linked with each other.	**False**	51	73.9	135	81.3	186	79.2	0.212
	The risk of suffering from arteriosclerosis is not hereditary.	**False**	41	59.4*	125	74.9*	166	70.3	0.028
	Arteriosclerosis can cause renal damage.	**True**	53	76.8	124	74.7	177	75.4	0.869
	With arteriosclerosis, blood platelets accumulate on the arterial walls.	**True**	54	78.3	117	70.5	171	72.8	0.261
	With arteriosclerosis, fat accumulates on the arterial walls.	**True**	45	75.2*	133	79.6*	178	75.4	0.030
	Medication can remove completely sediments from the arteries.	**False**	45	65.2	118	71.1	163	69.4	0.438
	With arteriosclerosis, arteries become brittle.	**True**	50	72.5	109	65.3	159	67.4	0.351
	A stroke is caused by artery obstruction.	**True**	51	73.9	139	83.2	190	80.5	0.107
	The nutrient supply to the brain is not affected by a stroke.	**False**	44	63.8*	142	85.5*	186	79.2	<0.001
	A stroke is characterized by a sudden dysfunction of the heart.	**False**	44	63.8	89	53.3	133	56.4	0.151
	A stroke is caused when overexcited cells produce too much electricity.	**False**	44	63.8	103	61.7	147	62.9	0.883
	Individuals with diabetes are more likely to suffer a stroke.	**True**	47	68.2	122	73.1	169	69.1	0.526
	Smoking is a minor risk factor with respect to a myocardial infarction.	**False**	35	51.5*	109	66.1*	144	61.8	0.031
	The oxygen supply to the heart is not affected by a myocardial infarction.	**False**	45	66.2*	138	83.1*	183	78.2	0.008
	Damage caused by a myocardial infarction is not usually permanent.	**False**	45	66.2*	133	80.6*	178	76.4	0.027
	Diabetes is a predisposing factor for a myocardial infarction.	**True**	42	60.9	122	73.5	164	69.8	0.062
	When suffering a myocardial infarction, pain may radiate into the stomach.	**True**	38	55.1	105	63.3	143	60.9	0.245
	A myocardial infarction can manifest itself through nausea and vomiting.	**True**	42	60.9	94	56.6	136	57.9	0.565

*p < 0.05.

**Table 4:** **Students’ response to the MetS questionnaire by gender -** ***poor*** **level of knowledge – (percentage of students answered correctly ≤50%)**

Conditions	Question	Correct answer	***Males (N = 69)***		***Females (N = 171)***		***Total (N = 240)***		***P Value***
			***n***	***%***	***n***	***%***	***n***	***%***	
**Diabetes**	Individuals with diabetes must have insulin shots.	**False**	30	43.5	76	44.7	106	44.4	0.887
	With diabetes, sugar cannot move in the blood.	**False**	25	36.2	56	32.8	81	33.8	0.652
**Adiposity**	The terms ‘overweight’ and ‘adiposity’ are synonyms.	**False**	35	50.7	70	41.7	105	44.3	0.250
	Liposuction is the best possible treatment in adiposity therapy.	**False**	27	39.1	75	44.6	102	43.0	0.472
**Hypertension**	People with hypertension are as likely to suffer from arteriosclerosis as those with normal hypertension.	**False**	32	47.1	67	39.2	99	41.4	0.309
	Pregnant women are as likely to suffer from hypertension as non-pregnant women.	**False**	31	45.6	76	44.7	107	45.0	1.000
**High serum holesterol**	High serum cholesterol does not cause acute ailments.	**True**	24	34.8*	26	15.7*	50	21.3	0.002
	Fatigue is a frequent symptom of high serum cholesterol.	**False**	16	29.2	30	18.1	46	19.6	0.372
**Arteriosclerosis**	With arteriosclerosis, arteries contract.	**False**	21	30.3	43	25.6	64	27.0	0.511
**Stroke**	A stroke is preceded frequently by chest pains.	**False**	32	46.4	80	47.9	112	47.5	0.886
**Myocardial infarction**	A myocardial infarction is caused by cerebral dysregulation of the heart.	**False**	32	47.1*	47	28.3*	79	33.8	0.009
	A myocardial infarction must be treated surgically.	**False**	25	36.3	60	36.1	85	36.2	1.000
	A myocardial infarction is typically followed by some degree of paralysis.	**False**	23	33.3	78	47.0	101	43.0	0.061
	A myocardial infarction is caused by malfunction of one or more heart valves.	**False**	14	20.3	24	14.6	38	16.2	0.331
	A myocardial infarction is usually preceded by loss of sensation and numbness.	**False**	20	29.0	33	19.9	53	22.6	0.161

*p < 0.05.

### Good level of knowledge of conditions relevant to MetS

Students indicated *good* knowledge on various questionnaire items about conditions relevant to MetS. As shown in Table 2, more than 80% of students were aware about diabetes types, eye complications, and increased risk in pregnancy. Ninety two percent of the students identified adiposity as a risk factor for heart disease and 84% knew that adiposity, and high serum cholesterol, can predispose individuals to arteriosclerosis. Likewise, more than 85% of students knew that individuals with hypertension may complain of dizziness and that high serum cholesterol can be treated with diet and medications.

Arteriosclerosis was clear to many students in regard to disease characteristics, symptoms, and risk factors. For example, 86% of the students correctly identified leg pain as a symptom of arteriosclerosis, 93% (85.5% of males and 96% of females) knew that arteriosclerosis increases the risk of suffering a stroke (*p* < 0.05), and 88% knew that a stent can be surgically inserted into an arteriosclerotic artery to increase the flow of blood through it.

As for the stroke questions, most students indicated good knowledge about causes, symptoms and complications of stroke with few statistically significant differences between genders. For example, 94.5% of the students knew that stroke affects the brain, but more females (97%) than males (88%) responded correctly to this question (*p* < 0.05). Also, 95% of the female students knew that a permanent speech defect is a potential consequence of stroke compared to 85.5% of the males (*p* < 0.05). Likewise, 89% of the females knew that a stroke is often followed by a memory dysfunction compared to 75% of males (*p* < 0.05) and 86.4% of the students were aware that a stroke is usually preceded by speech problems.

Students indicated good knowledge about myocardial infarction in terms of causes, symptoms and treatment with few gender differences. For example, 85% of students knew that obstructive coronary artery disease may cause myocardial infarction and 91% of students knew that most myocardial infarctions can cause chest pain radiating into the right or left arm. More females (92%) than males (79%) knew that shortness of breath may precede a myocardial infarction (*p* < 0.05) which may lead to permanent damage of the affected heart tissue (85% of females vs. 75% of males), and 89% of the students were aware that anticoagulant medications are administered after a myocardial infarction.

### Fair level of knowledge of conditions relevant to MetS

Table 3 shows the questions where students’ correct answers were between (51-80%) indicating fair knowledge. Students showed fair knowledge about diabetes in regard to risk factors, symptoms, diet and complications. About 80% of students knew that hereditary factors can play a role in the development of diabetes. Polyuria was identified by 74% of students as a common symptom of diabetes and 64% identified arteriosclerosis as one of the sequela of diabetes. However, 45% of the students (58% of males and 39% of females) falsely believed that patients with diabetes can only eat special kinds of sweets (*p* < 0.05).

Adiposity was fairly understood by students in regard to risk factors and treatment. About 69% of students were aware that obesity increases the chance of suffering a stroke and 73% knew that adiposity can be treated surgically. As for hypertension, students indicated fair knowledge in terms of risk factors, causes, and treatment. About 80% of students were aware that hereditary factors can contribute to hypertension. However, 43% of students falsely believed that most cases of hypertension can be attributed to a single, easily identified cause, and 33% assumed that antihypertensive medications could be discontinued once blood pressure was under control. Complications of untreated hypertension, such as kidney and eye diseases, were identified by 70% of students.

### Poor level of knowledge of conditions relevant to MetS

Table 4 shows the questions where less than 50% of students answered the questions correctly, thus indicating *poor* knowledge or areas for improvement. Students showed poor knowledge about some aspects of the physiology and treatment of diabetes. About 66% of the students assumed that sugar cannot “move” in the blood with diabetes and 56% thought that all patients with diabetes must take insulin injections. In regard to adiposity, 56% of students thought that the terms “overweight” and “adiposity” were synonymous and 57% of students falsely believed that liposuction was the best possible treatment for adiposity.

In terms of hypertension, only 45% of students knew that pregnant women were more likely to suffer from hypertension than non-pregnant women. Consequences of high serum cholesterol were not clear to students as 79% thought that high serum cholesterol causes acute ailments and 80% of students falsely believed that fatigue could be a frequent symptom of high serum cholesterol. There were few false beliefs among students about arteriosclerosis, stroke, and myocardial infarction. For example, 73% of students falsely believed that with arteriosclerosis, arteries contract and 52.5% of the students falsely believed that a stroke is preceded by chest pain.

Myocardial infarction was not clear to students in terms of causes, symptoms, and treatment. About 66% of students falsely believed that a myocardial infarction is caused by cerebral dysregulation of the heart, 84% thought that myocardial infarction is caused by malfunction of one or more heart valves, 77.5% assumed that myocardial infarction is preceded by a loss of sensation and numbness, 57% believed that myocardial infarction is typically followed by some degree of paralysis. More than two-thirds of the students (64%) thought that myocardial infarction must be treated surgically.

### Total score in relation to gender, major of study, year in school, family history, BMI, and ethnicity

Results of the Poisson Regression model regressing total score on gender, major of study, any family history, overweight/obese, ethnicity, and year in school as covariates, indicated that gender, major of study and year in school were significant factors associating with differences in total score. Controlling for covariates mentioned, females scored 6% higher than male students (p = 0.01), Health Science major students scored 7% higher (p = 0.0011) than non- Health Science major students. Compared to 1^st^ year students, 5^th^ year had 11% higher scores (p = 0.002). Family history of disease, BMI and ethnicity were not significantly associated with total score (Table 5). Table 6 shows the average percent correct answers per condition, which ranged from 62.7% (lowest percentage on adiposity questions) to 77.3% (highest percentage on stroke questions).

**Table 5:** Poisson Regression Model - Total Score*

	Total score	
**Variable**	OR*	***P*** **Value**
Gender (Female)	**1.06**	**0.010**
Health sciences major	**1.07**	**0.001**
No family history	0.97	0.106
BMI <25	0.99	0.530
Ethnicity (White)	1.05	0.111
2nd year	0.98	0.557
3rd year	1.00	0.927
4th year	1.06	0.059
5th year	**1.11**	**0.002**

*overall model *p* < 0.001.

*OR (odd ratio).

**Table 6:** **Summary of Average Scores and Percentage of Correct Answers Per Condition as Questionnaire Topic Areas (N = 243)**

Condition	Maximum score	Average score	SD*	Average percentage of correct answers	SD*
Diabetes	16	10.7	2.5	66.6	15.9
Adiposity	9	5.7	1.6	62.7	17.4
Hypertension	12	8.3	2.4	68.8	20.2
Cholesterol	6	3.8	1.1	62.7	18.5
Arteriosclerosis	17	12.6	3.2	74.1	18.7
Myocardial infarction	18	11.7	3.0	64.8	16.8
Stroke	12	9.3	2.6	77.3	21.5

* Standard deviation.

**Figure 1:**
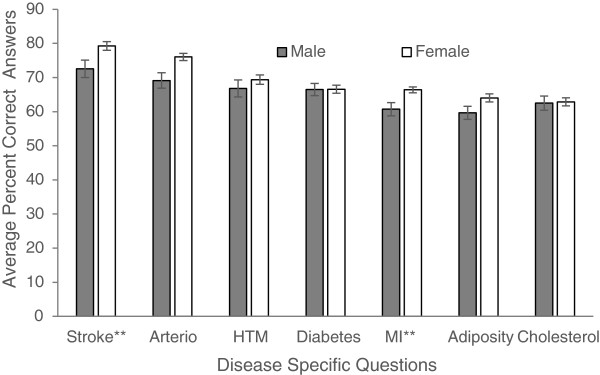
**Students’ knowledge based on gender.**

**Figure 2:**
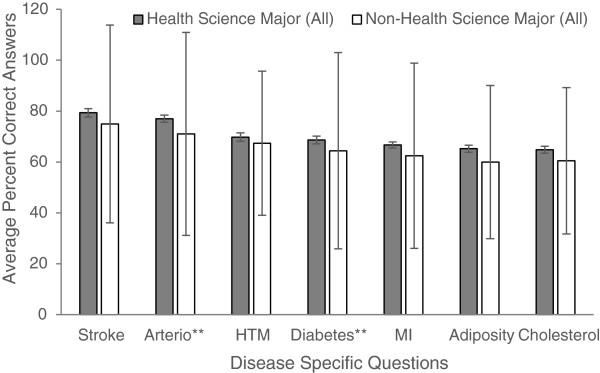
**Students’ knowledge based on major of study.**

**Figure 3:**
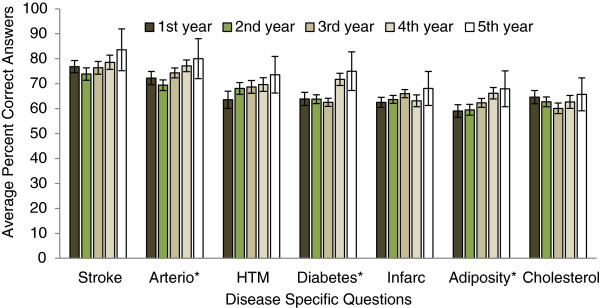
**Students’ knowledge based on year in school.**

**Figure 4:**
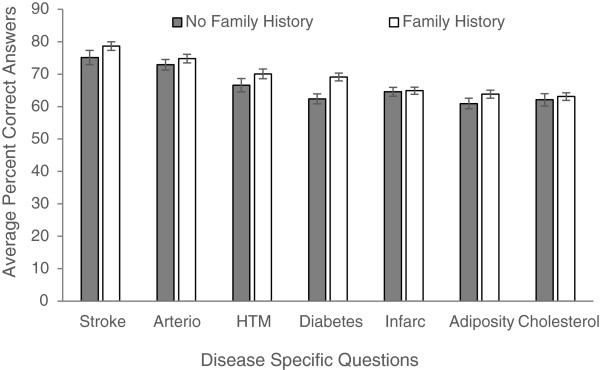
**Students’ knowledge based on family history.**

**Figure 5:**
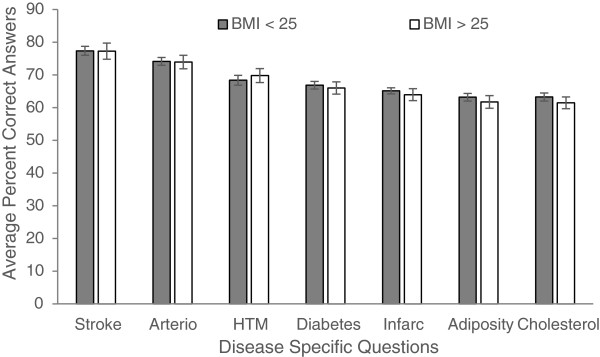
**Students’ knowledge based on weight status (healthy weight vs. overweight/obese).**

Figures 1, 2, 3, 4 and 5 explore visually the trends for average percent correct answers per condition for various characteristics. Females scored higher than males on most conditions, except for “Diabetes” and “Cholesterol” sections, where males had similar average score as females (Figure 1). Health Science major students consistently scored higher than non-Health Science major students (Figure 2). Those students in the 5^th^-year of study scored higher than those students in the 1^st^, 2^nd^, 3^rd^, or 4^th^- years; however, there was an overlap between the other grade years (Figure 3). While those students with family history of some diseases generally scored higher than those students with no-family history, this difference was not statistically significant for most of the tests (Figure 4). There was no difference between the knowledge of healthy weight students and that of obese or overweight students (Figure 5).

## Discussion

Study’s results show that students were most knowledgeable about arteriosclerosis and stroke conditions and least knowledgeable about adiposity and cholesterol. There were some false beliefs held by students. For example, 56% of the students falsely believed that all individuals with diabetes must take insulin injections, 55.4% falsely believed that individuals with diabetes can only eat special kinds of sweets, and 57% falsely identified liposuction as the best possible treatment for adiposity. There was no pattern for significant gaps in knowledge between the conditions or type of knowledge missing. The average percentage correct answers by condition varied from 62.7% to 77.3%. Similar analyses by type of information (enabling conditions often leading to, description of physical changes, outcomes of condition and treatment of the conditions) did not reveal any consistent gaps in knowledge. Therefore, the discussion of areas for improvement will be explored by the individual questions. As expected, Health-Science major students scored 7% higher than non-Health-Science major students (*p* < 0.001), and 5^th^- year students scored 11% higher than 1^st^-year students (*p* < 0.001). Students majoring in Health Sciences would have covered these topics more intensely than students of non-Health Science majors. Also, students in their 5^th^-year would be expected to perform better than 1^st^-year students as they are in their final year of schooling and would have covered more coursework on the topic. In regards to gender, female students showed better “understanding” since being female was associated with a 6% higher score after controlling for the other covariates in the regression model (*p* < 0.01). Female students scored higher than male students on most questionnaire sections, except for “Diabetes” and “Cholesterol” sections, where scores were similar for both genders. It could be argued that the gender differences seen in this study were due to the *field of study* as more females (58%), than males (36%), were majoring in Sciences; however, based on the Poisson Regression analysis, after controlling for covariates (major of study, year in school, BMI, ethnicity), females still scored higher than male students (*p* < 0.01). One possible explanation for this difference in results might be that female students are more concerned about their body shape and may have more interest in health issues than male students at college age [36]. Male students, in general, have more interest in extracurricular activities than in health issues. Results also indicated that students with family history of some diseases generally scored higher than students with no family history, but differences were not statistically significant. To test whether students’ specific family history was associated with knowledge by score condition, (for example if students had a family history of diabetes, were their diabetes scores higher), regression analysis did not indicate any significant differences.

Nevertheless, in spite of the majority of students correctly identified symptoms and complications of diabetes, adiposity, hypertension, arteriosclerosis, myocardial infarction and stroke, there were some misconceptions held by students on questionnaire items that have implications for future health education efforts and whether students will be prompted to make personal healthy lifestyle choices. For example, in terms of diabetes, about half of the students believed that individuals with diabetes can only eat special kinds of sweets and more than two-thirds of the students thought that sugar cannot “move” in the blood in patients with diabetes. These results reflect a lack of understanding about the etiology and treatment of diabetes. The notion that individuals with diabetes cannot eat sweets or desserts like anyone else may also reflect the general public’s notions about diabetes. Diabetes is a serious health problem and it is the seventh leading cause of death in the United States [37]. Thus, educating students about this disease as a first step in awareness and knowledge is important as a precursor to other health education efforts that could lead to actual behavior changes to reduce their personal risk of developing diabetes or metabolic syndrome later in life.

Central obesity is a leading risk factor for metabolic syndrome. Results indicated that the majority of students, irrespective of weight, were aware that adiposity is a risk factor for heart disease, stroke and arteriosclerosis. However, students were not clear about whether the terms “overweight” and “adiposity” are synonyms. These terms are often misleading and sometimes used as synonyms, even though the former refers to the gaining of body weight, and the latter refers to an excess of body fat. Also, adiposity treatment was not clear to students as more than half of the students thought that liposuction is the best possible treatment in adiposity therapy. This finding may be related to how the media portray liposuction as a revolutionary treatment for adiposity therapy. It was reported that most college students acquire their health information via television, popular magazines, and the internet rather than from credible, evidence-based information sources [38]. Confusion about overweight and adiposity and the notion that liposuction is the best possible obesity treatment may contribute to students failing to understand that the amount and placement of adipose tissue in the body are more important concepts than body weight itself. While the survey did not specifically measure knowledge related to body fat distribution, e.g. central obesity, lack of clarity about adiposity may further confuse the issue. The belief that liposuction is the best possible obesity treatment, may lead to students overlooking the importance of making healthy lifestyle choices, including healthy eating habits and regular physical activity, as the primary prevention and treatment options. Also, this could lead students to the perception that healthy lifestyles are not necessarily important since other actions can be taken later to resolve the health consequences.

In this study, findings related to the hypertension questions indicated that the majority of students knew that hypertension can be manifested as dizziness, is linked to the genetic makeup of an individual, and heredity is a risk factor for this condition. However, when students were asked whether pregnant women are as likely to suffer from hypertension as non-pregnant women, only 45% of the female students responded correctly to this question. In addition, only 41.4% of the students knew that there is a relationship between hypertension and arteriosclerosis. Hypertension is often called a “silent” killer because it usually starts with no symptoms or warning signs and progress before individuals realize that they have it. It has been estimated that about 1 in 5 (20.4%) adults in the U.S. have high blood pressure but are unaware of it [39–42]. In this regard, students’ correct response identifying that dizziness is a symptom of blood pressure may lead them to a false sense of safety if they wait for this sign and symptom, prior to seeking health advice or making healthy lifestyle choices, to prevent the development of hypertension. Students’ response also indicates a lack of understanding about risk for hypertension during pregnancy and the connection between hypertension and arteriosclerosis.

In regard to the cholesterol questions, results are encouraging as more than two-thirds of the students were aware that family history is a risk factor for high serum cholesterol, and more than 85% of the students were aware that high serum cholesterol can be treated with diet and medication. In 2008 in Germany, Becker et al. reported that only about half of their participating students knew that heredity and serum cholesterol were related, and that high serum cholesterol can promote arteriosclerosis [29]. The differences seen in students’ responses in our study and that of Becker et al. might be due to the cultural differences in risk perceptions. It might be that our students are more familiar with cholesterol as a risk factor for heart disease than students in the Becker et al. study [29] since heart disease is the number one killer in the U.S. [6].

As for arteriosclerosis questions, 93% of students were aware that arteriosclerosis can increase the risk of suffering a stroke, but more than two-thirds of the students were not aware that elevated cholesterol can thicken the wall of the arteries leading to stiffness and loss of elasticity, and only 20% knew that high cholesterol did not cause “acute” ailments and that fatigue was not a symptom of elevated cholesterol. Therefore, it is unclear that the ultimate connection between elevated serum cholesterol, arteriosclerosis, and stroke is fully understood by students. Morrell et al. conducted a study among 360 undergraduate students recruited from three universities in the U.S. and reported that 12% of their male participants had MetS compared to 6% of their female participants [22]. In general, men do not routinely seek medical care and discuss health complications with their healthcare providers, and in this study also male students showed lower knowledge about these conditions [43]. This suggests that there is a need to increase knowledge of the interconnections between elevated cholesterol and CVD complications among students, especially male students. This lends support for campaigns such as “*know your number*” [44] as an effective way to raise awareness among male students about the need to have their serum cholesterol measured and for each student to know his results in order to spur the necessary lifestyle changes, if needed.

As for myocardial infarction and stroke questions, there seems to be confusion between myocardial infarction and stroke as almost half of the students believed that a stroke was frequently preceded by chest pain and is characterized by a sudden dysfunction of the heart. More than two-thirds of the students falsely thought that myocardial infarction is caused by cerebral dysregulation of the heart, and 84% believed that myocardial infarction is caused by malfunction of the heart valves. Similar findings were reported by other authors. Becker et al. [29] reported that more than one-third of their participating students thought that a stroke often begins as chest pain and about 70% believed that myocardial infarction was due to malfunctioning of the heart valves [29]. However, symptoms of myocardial infarction were better understood by students as the majority of students, especially females, knew that most myocardial infarctions can cause chest pain that can radiate into the right or left arm, shortness of breath, and can permanently damage the heart tissue. However, over 70% of the students falsely believed that a myocardial infarction was usually preceded by a loss of sensation and numbness, more than half of the students inaccurately believed that surgical treatment was needed, and that paralysis was an outcome of myocardial infarction. Munoz et al. [38] also reported that a high proportion of their female students knew the causes of heart disease and were aware of the basic warning signs associated with a heart attack [38].

Whether specific knowledge about the individual conditions and signs and symptoms is critical to students to recognizing their *personal* risk and making the behavior and lifestyle changes at an early age that are necessary to prevent these conditions is still unknown. However the results of this study indicate that there are opportunities for clarifying understanding about specific conditions. In particular, health education may need to be developed that targets students of non-Health Science majors since their knowledge is different than Health Science majors. The level of understanding is only significantly different for those students in the 5^th^ year of school. Educating students about cardiovascular diseases and stroke at early stages is very important; especially risk factors for heart disease that are established at an early age [45].

As indicated previously, the impact of a Science-based curriculum on health knowledge is confirmed as expected by these study results and this increased knowledge cannot be attributed to the higher proportion of females, versus males, that reported a Science field of study. As students become more educated about MetS risk factors, positive lifestyle changes are more likely to be followed. Colleges and universities should put their emphasis not only on students’ mainstream coursework but also on students’ health education that involve preventive strategies to promote good health and minimize the development of MetS risk factors among students at an early age. It has been documented that lifestyle modifications are an effective treatment strategy to reduce MetS [46–51]. In this essence, mandating the incorporation of health education courses into the curriculum should be a priority for colleges and universities to enhance students’ wellness and promote a healthy lifestyle that nurtures students’ academic success - in particular for non-Health Science majors since they have the lowest level of knowledge.

### Study’s strengths and limitations

Given the serious health problems resulting from MetS, evaluating students’ level of awareness and knowledge about conditions relevant to MetS is crucial. Colleges and universities are often the final academic settings where students can acquire knowledge about health-related issues and develop positive lifestyle skills. Findings from this study will help in filling the gap in evaluating students’ knowledge about conditions relevant to MetS and in identifying students’ existing false beliefs. Critical baseline information was provided in this study and results can be used in developing health education courses tailored to students’ needs. The main limitation of this study is the small sample size, which limits the generalization of this study’s findings and limits the statistical power to detect significant differences between genders. Therefore, this study’s results should be interpreted with caution, as results may not be generalizable to all university students. Another limitation is the use of the cross-sectional design which cannot distinguish whether the false belief is a result of students’ lack of awareness or due to students’ pre-existing knowledge. Also, this study was limited in that most of the participants were female students (72% female vs. 28% male). Female students may have been more interested in research related to health issues than male students, since participants voluntarily entered into the study. Nevertheless, the dominance of female participants reflects the university’s student body data and is consistent with the gender composition of a large-scale national study [35]. The participants’ answers were self-reported; thus, factors such as question interpretation and understanding by students may have also affected the results. Lastly, it would be important to replicate this study in schools having a more ethnically diverse student population than in the current study and to include students pursuing a greater variety of majors. Given the serious implications of MetS on health, research on this topic deserves further attention.

## Conclusions

The present study provides insight into students’ knowledge about conditions relevant to MetS.

Students’ understanding of conditions relevant to metabolic syndrome differed among domains, with few gender differences and few false beliefs. Knowledge about a health condition’s signs and symptoms, health consequences, and treatment are prerequisites to the next component of the health belief model—understanding “*your*” *personal* risk. While the students acknowledged the importance of heredity, this may still not translate into *personal* risk for them at the level necessary for them to consider behavior change and adoption of more healthy lifestyles. In this study, *specific family history* did not impact students’ knowledge in those *specific conditions*. Thus, using family history as a perceived threat (according to Health Belief Model) to motivate students may not be effective. This research indicates that health education programs at the college level may be able to assume basic knowledge about the various conditions related to MetS; however, there is a room for improvement. Also, colleges may need to focus on the how these conditions relate to the students on a *personal* level, so that this knowledge is more likely to lead to healthy lifestyles. A recent study conducted by Hawkins et al. indicated that knowledge acquisition via education is effective when education is tailored to students’ needs [52]. Other researchers also noted that acquiring knowledge about health-related problems was associated with more positive changes [53, 54]. In this essence, health education based on individual’s “*personal*” risk is important to facilitate voluntary adaptions of healthy behavioral choices.

Given the increasing prevalence of MetS among college-aged adults, raising awareness about MetS among this age group is important to reduce the prevalence of this condition. Colleges and universities are ideal settings to educate students about health issues, however, they need to move beyond simple knowledge acquisition and focus on the ability to connect this knowledge on a “*personal*” level to the individual student’s perception of their own risk and then equip them with the right skills needed to help them translate knowledge into positive lifestyle behaviors. Previous studies have indicated that lifestyle interventions, including a healthy eating pattern, regular physical activity, and weight management, can substantially reduce the prevalence of MetS among various age groups of different populations [42–46]. Future research should examine whether students’ knowledge of conditions relevant to MetS did translate into their lifestyle practices towards Mets risk reduction. This question can be investigated by assessing the actual prevalence of MetS among CMU students. Future research should also explore the strategies that can stimulate students’ *personal* risk perception to create positive change in their lifestyle behaviors.

## Abbreviations

MetS: Metabolic Syndrome
CVD: Cardiovascular Disease
NHANES: National Health and Nutrition Examination Survey
HDL-C: High Density Lipoprotein-Cholesterol, BMI, Body Mass Index.
